# Supplementary material for: Embryogenic Stem Cell Identity after Protoplast Isolation from Daucus carota and Recovery of Regeneration Ability through Protoplast Culture
Source: Int J Mol Sci. 2022 Sep 30;23(19):11556. doi: 10.3390/ijms231911556 (PMC9570137; doi:10.3390/ijms231911556)
Supplement: Supplementary file 1 [file ijms-23-11556-s001.zip › ijms-1935515-SI.pdf]

Table S1. Composition of the reagents and media used in this study.

Reagent	Composition	Sterilization
CPW solution	1 mM $CaCl_2 \cdot 2H_2O$, 0.4 M mannitol, 5 mM MES	Autoclave
Digestion solution	CPW + 1.0% Viscozyme® L[x] + 0.5% of Celluclast® 1.5 L[y] + 0.5% Pectinex® XXL[z]	0.2 μm syringe filter
W5 solution	154 mM NaCl, 125 mM $CaCl_2 \cdot 2H_2O$, 5 mM KCl, 2 mM MES	Autoclave
Sodium alginate solution	0.4 M mannitol, 130 mM sodium alginate	Autoclave, 0.2 μm syringe filter
$CaCl_2$-agar	0.4 M mannitol, 20 mM $CaCl_2 \cdot 2H_2O$, 1.0% plant agar	Autoclave
$CaCl_2$ solution	0.4 M mannitol, 50 mM $CaCl_2 \cdot 2H_2O$	Autoclave
**Media**	**Composition**	**Function**
Protoplast culture medium (PCM)	MS (including vitamin), 0.45 μM 2,4-D, 0.91 μM zeatin, 0.4 M glucose, 0.025% casein hydrolysate	Induction of protoplast division
Plant regeneration medium (PRM)	MS (including vitamin), 0.08 M sucrose, 0.26% gelrite	Plant regeneration

[X]Viscozyme® L: Multi-enzyme complex containing a wide range of carbohydrases, including arabanase, cellulase, *β*-glucanase, hemicellulase, and xylanase. (Novozymes, Bagsværd, Denmark)

[Y] Celluclast® 1.5 L: Cellulase complexes with *β*-glucosidase for degrading cellobiose to glucose polymer. (Novozymes, Bagsværd, Denmark)

[z] Pectinex® XXL: Pectinases complexes with hemicellulases and arabinanases. (Novozymes, Bagsværd, Denmark)

Table S2. Primer sequences for the RT-qPCR analysis.

Gene	Direction	5′ → 3′	Amplicon size (bp)
DcLEC1	Forward	TCTGGGCTTTGACGACTACA	136
	Reverse	GGTGCTCAAGTGACCTCCTC	
DcBBM	Forward	ACTTTCAGCAGCACCCTTGT	117
	Reverse	GTGCAGGGAGGTTGAGAGAG	
DcWUS	Forward	GTTGGTCATCACATGGCAAG	109
	Reverse	TGTTGGAGATTCCACTGCTG	
DcDRN	Forward	GAAATCTCCGCAAGCAAAAC	106
	Reverse	ATCAGACGGCTCTGGAGAAA	
DcSERK	Forward	TGGCGGTTAAAAGGCTAAAA	142
	Reverse	AGTAGCCGCTCAGTTGGTGT	
DcActin	Forward	GCGGGAAATTGTTCGTGATATG	182
	Reverse	TAGATGGCTGGAAGAGGACTT	
